# An educational intervention to implement skin-to-skin contact and early breastfeeding in a rural hospital in Mexico

**DOI:** 10.1186/s13006-019-0202-4

**Published:** 2019-02-06

**Authors:** Luis Fernando Sanchez-Espino, Gregorio Zuniga-Villanueva, Jose Luis Ramirez-GarciaLuna

**Affiliations:** 1 Pediatrics Department, Tecnológico de Monterrey, Batallón de San Patricio 112, Real de San Agustín, 66278 San Pedro Garza García, Nuevo León Mexico; 2 Pediatrics Department, Tecnológico de Monterrey, San Pedro Garza García, Nuevo León Mexico

**Keywords:** Skin-to-skin contact, Early breastfeeding, Rural hospital, Educational intervention

## Abstract

**Background:**

Early skin-to-skin contact promotes infant physiologic stability, provides warmth and makes breast milk readily available. Despite the known benefits of early skin-to-skin contact, this practice is not included within standard care in the Mexican public healthcare system. After birth, newborns are usually taken to an incubator in the nursery where they transition to extrauterine life and receive either dextrose 5% or infant formula for their first feed. The aim of this study was to assess if a dual educational intervention in a rural hospital in Mexico could modify current practice and accomplish early skin-to-skin contact and early breastfeeding.

**Methods:**

A two-step educational intervention was designed. The first step was to educate the labor and birthing staff of the hospital, and the second step was to educate all pregnant women with uncomplicated pregnancies at 36 weeks’ gestation. The educational intervention explored the benefits, implications and steps of early skin-to-skin contact and early breastfeeding. All births were registered for the three month period following the intervention. The time of onset of skin-to-skin (SSC) contact, its duration and time of initiation of breastfeeding were recorded and analyzed using ANOVA testing.

**Results:**

A total of 142 births met our inclusion criteria, from those, 77% (*n* = 109) received skin-to-skin contact and early breastfeeding. The average time of initiation of skin-to-skin contact in the first and last month of the study was 18.5 (± 2.2) and 9.6 (± 2.2) minutes of life, respectively (*p* < 0.001). The average duration of SSC in the first and last month was 22 (± 10.9) and 40.9 (± 17.4) minutes, respectively (*p* < 0.001). The average time of onset of breastfeeding in the first and last month was 48.9 (± 15) and 34.4 (± 16.7) minutes of life, respectively (*p* < 0.001).

**Conclusions:**

A simple and low-cost educational intervention achieved the inclusion of skin-to-skin contact and early breastfeeding as part of standard care in a rural hospital. Further studies could replicate our intervention in similar settings to test the generalizability of the findings.

## Background

Skin-to-skin contact (SSC) is defined as the placement of the newborn over the mother’s bare chest at the time of birth for at least one hour [1], where breastfeeding can be initiated immediately [1]. It is classified according to the time of onset as immediate if achieved within the first 10 min of life, or early contact if achieved between the first 10 min and 24 h of life [2].

Multiple benefits have been documented for both infants and mothers when exposed to SSC, such as decreasing infant mortality rates, facilitating the transition to extrauterine life [3], promoting thermoregulation and early hemodynamic stability even in premature babies, promoting metabolic balance and optimal neurological development, decreasing the incidence of postpartum depression, reducing infant crying, improving mother and infant bonding [4, 5], and increasing maternal oxytocin levels [6].

One of the most studied benefits of SSC is its impact on breastfeeding, which is a crucial practice for maternal and child health [7]. Skin to skin contact facilitates early breastfeeding and improves latching [6], increases the mother’s milk production and supply, and has been associated with both longer exclusive breastfeeding times for the first six months of life, and overall longer breastfeeding duration, which includes the interval of time after the first six months of life [8].

Despite the known benefits of early SSC, this practice is still not included within the Official Mexican Standard NOM-007-SSA2–2016 entitled *“For the care of the woman during pregnancy, birth and puerperium, and of the newborn”* (NOM-007) [9]. The Mexican NOM-007 stipulates that breastfeeding should be initiated within the first 30 min of life, but unfortunately, it has been documented that up to 70% of hospitals in Mexico do not offer breastmilk to newborns in their first feed due to cultural practices that have been erroneously transmitted historically [10]. Rather than offering breast milk right after birth, most infants are taken to the hospital’s nursery where they transition to extrauterine life and receive either dextrose 5% or infant formula for their first feed. The newborns are kept in the nursery for at least two hours and then they are taken to their mother, where they are finally breastfed for the first time but without SSC [10]. Since these practices can probably be modified by educating the healthcare practitioners, this study aimed to assess the impact of an educational intervention to modify the current practice and promote early SSC and early breastfeeding in a rural hospital in Mexico.

## Methods

### Setting

This study was carried out in a public second-level hospital in the rural community of Montemorelos, in the state of Nuevo Leon in Mexico during 2016. Montemorelos is a community of 60, 829 inhabitants [11] that gave birth to 2111 live newborns in 2015 [12], obtaining an estimated birth rate of 28.81 per 1000 inhabitants. From the total births of that year, 972 (46%) took place at the study hospital.

The hospital’s labor and birthing area consist of two adjacent rooms with three beds for labor, one birthing bed, one infant radiant warmer, and one recovery bed for the immediate puerperium. As a second-level hospital, all high-risk pregnancies and all women in preterm labor (< 37.0 weeks of gestation) were referred to another tertiary level hospital in the city of Monterrey, Nuevo León, Mexico, thus only non-complicated full term pregnancies were included in the sample.

### Routine care before our study

Before our intervention, the customary practice after birth was to place the newborn in the infant radiant warmer where they were dried, stimulated and assessed by either a pediatrician, a pediatric resident or a medical intern under supervision. Apgar scores were recorded during this time. After determining that the infants required no further reanimation, the nursing staff performed ophthalmic prophylaxis, application of vitamin K, identification of the child with a bracelet, obtained the fingerprints for the birth certificate, and recorded their weight and measurement. Afterwards, the newborns were wrapped in a warm cloth and were briefly introduced to their mothers before they were taken to the nursery. At the nursery, the babies were laid in an incubator where they stayed for at least two hours as they transitioned to extrauterine life. While in the incubator, the neonates were given either dextrose 5% or infant formula for their first feed, depending on the attending physician’s preferred choice. After this period, they were taken out, bathed and dressed to finally be taken to their mothers’ side, where they would start breastfeeding. Like most hospitals in Mexico, our hospital has no midwives, hence, all activities, education and care are performed either by the nursing or medical staff. The lack of midwives in Mexico responds to their continued relegation during the twentieth century, which limited “traditional” midwives to practice only in indigenous communities and not as part of the healthcare system [13].

### Proposed change in practice

**Fig. 1:**
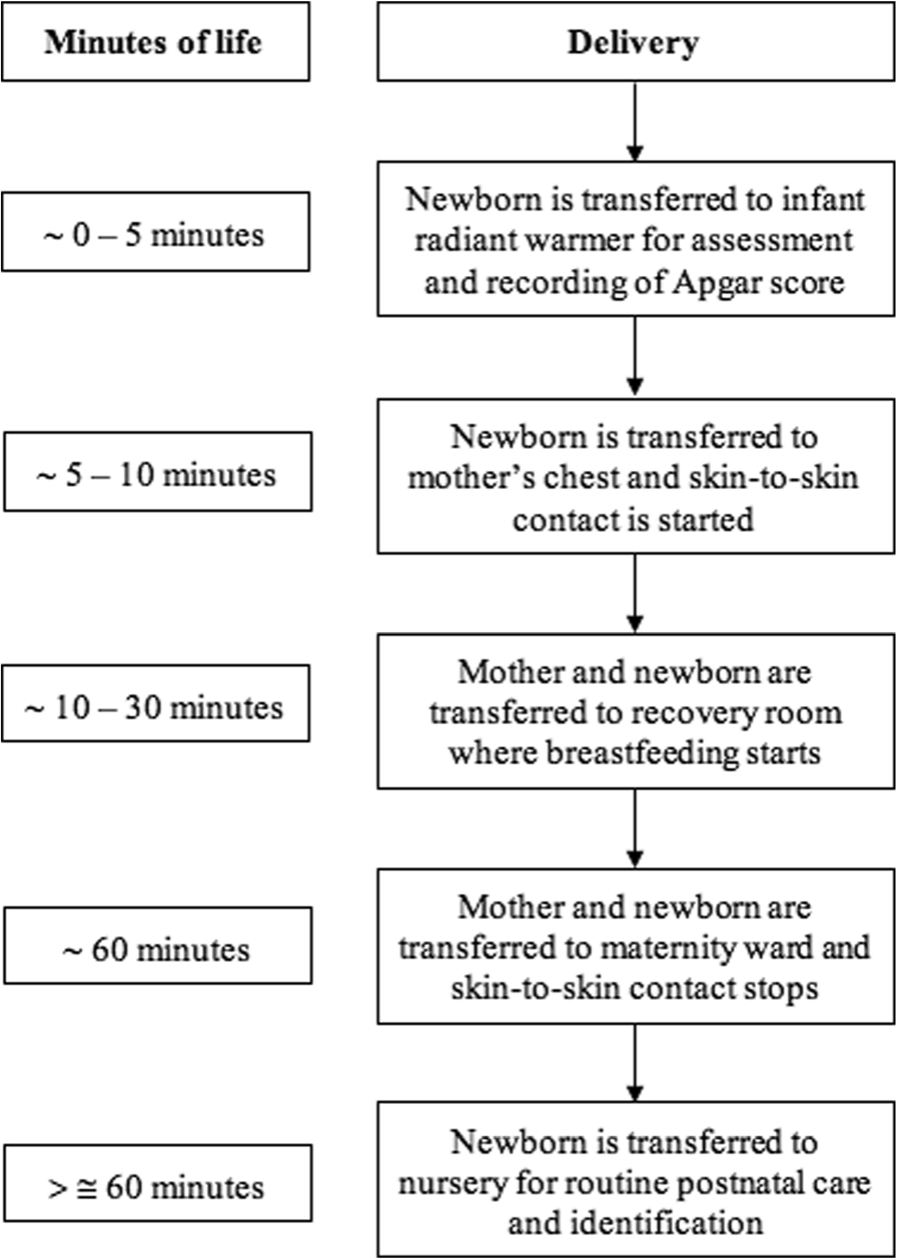
Skin-to-skin contact and early breastfeeding steps at our hospital

A thorough discussion with all the involved hospital departments was done before hand, and the study was approved by the hospital’s ethics and research committee. Given that this was a radical change in a practice that had been in place for years, our proposal was met with some resistance. Even though the procedure on how to perform SSC has been very well documented [14], a compromised decision was finally reached with an agreement upon the steps on how SSC and early breastfeeding could be performed at our site, with the goal of achieving immediate, continuous, and uninterrupted SSC as per the algorithm described by Brimdyr et al. [15]. The steps are described as follows (Fig. 1):

1. The attending pediatrician or pediatrics resident or intern under his supervision receives the newborn in the infant radiant warmer.
2. While being on the infant radiant warmer, the newborn is dried, assessed and examined. Apgar scores are determined within the first and fifth minute of life and recorded. (This means all newborns had a delay of up to five minutes before SSC was initiated.)
3. The naked newborn is then placed directly on the mother’s bare chest, with the mother’s hand holding the newborn’s back and both are covered with a sterile cloth for temperature regulation.
4. The newborn remains on the mother’s chest in the birthing room while the obstetrician finishes the mother’s post-birthing care.
5. The mother and the newborn are then transferred out of the birthing room into the recovery room without removing the newborn from the mother’s chest.
6. Once in the recovery room, with uninterrupted SSC, mothers are encouraged to initiate breastfeeding as soon as possible.
7. The newborn remains on the mother’s chest until both are transferred to the maternity ward, where the infant is finally separated from the mother and transferred to the nursery.
8. At the nursery, the ID bracelet is placed, the newborn is measured, weighed and bathed, and receives ophthalmic prophylaxis and application of vitamin K.
9. The newborn is finally dressed and brought in a crib to the maternity ward to allow rooming-in with the mother.

### Educational intervention

A two-step educational intervention was designed to institute the changes proposed. First, all staff members assigned to the labor and birthing service were trained by two senior pediatrics residents (GZ, AR). A total of 36 subjects (6 residents, 5 senior medical students, 7 nurses, 10 nursing interns, and 2 area directors) received a one-time 45-min lecture that covered the benefits and methodology of SSC and early breastfeeding and incorporated recommendations based on the baby friendly hospital initiative [16]. Sessions consisted of several training groups divided into one morning group, one evening group, three night-groups and one weekend group, covered within a one-week period.

The second part of the educational intervention consisted in providing education to the expecting mothers. Currently, all pregnant woman affiliated with the Mexican government’s healthcare insurance plan *Seguro Popular* are required to receive several educational sessions throughout their pregnancy covering different health-related topics. Routinely at our hospital, all women who reach 36 weeks of gestation are gathered to receive an educational session during that week, and these sessions are held on a weekly basis. For the second step of our intervention, we included during the session a 45-min lecture offered by the head of the pediatrics department (MV) where the study was introduced, and the benefits of SSC and early breastfeeding discussed. Time was allowed for the expecting mothers to ask any questions, and after the session, all women who attended were asked to participate in the study, and if agreed on, consent was obtained.

The techniques used during the lectures, for both health personnel and pregnant women, were audiovisual presentations through PowerPoint®.

### Timeline of the study

Pediatrics residents in Mexico perform a 4-month rotation in a rural hospital as part of their training during their senior year of residency. The study hospital’s education department asks that residents develop a quality improvement project during their rotation as part of their work. The present study was the result of such project undertaken by two senior pediatrics residents (GZ, AR) during their rotation from March 2016 to August 2016. During the first month of their rotation, the study was designed, and the first step of the educational intervention was performed. The second step of the intervention started on the last week of March and continued through the three remaining months of their rotation. The study was limited in time, as results needed to be presented by the end of their rotation. Thus, a follow-up time of three months was only feasible.

### Variables analyzed

The time of onset of the SSC, its duration and the time of initiation of breastfeeding from the moment of birth were recorded by nursing staff in the newborn’s chart. The data was extracted by one of the researchers (LS) and analyzed by another (JR). Variables were measured only in those births who received SSC and early breastfeeding, as the rest received standard care and the variables could not be measured. No information regarding breastfeeding initiation or time of onset before our study were available. The included population for the study were low risk full term pregnancies born through vaginal births (instrumented births included). Premature births, cesarean births, and newborns requiring advanced neonatal resuscitation were excluded.

### Statistical analysis

In light of the characteristics of the study, a descriptive analysis was performed to describe the characteristics of the births. Only those births who received SSC and early breastfeeding were analyzed using ANOVA tests with Tukey post hoc tests for multiple comparisons to assess change in the time of onset of the SSC, its duration and the time of initiation of breastfeeding through the three months of duration of the study. ANOVA tests were used to assess potential confounders (month of the study, the age of the mother, gravidity, and gestational age at birth). Statistical analysis was performed using the R software v. 3.0.2® [17] at the 95% confidence interval.

## Results

**Fig. 2:**
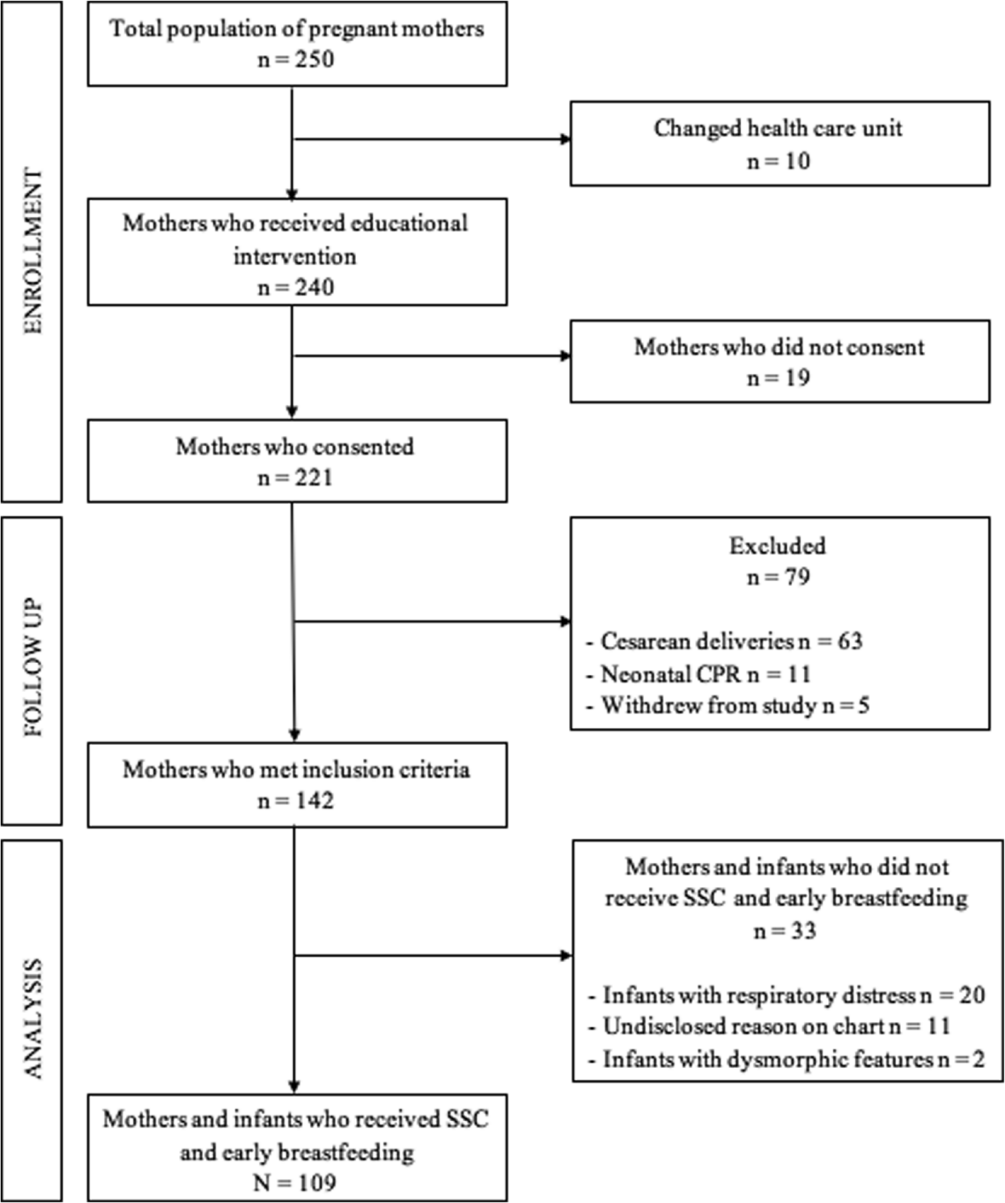
Selection of participants diagram

From the total number of pregnant mothers who were being followed during their pregnancy at our hospital (*n* = 250), 96% (*n* = 240) attended the educational session during their 36th week of gestation. The remaining 4% (*n* = 10) either migrated from Montemorelos or were transferred to a different healthcare unit before their scheduled session and had their birthing at a different hospital. Of those who were present for the educational intervention, 92% *(n* = 221) consented to participate in the study, the remaining 8% (*n* = 19) did not. A total of 79 births were excluded due to the following reasons: 63 (29% of mothers who consented) were cesarean births; 11 (5%) required neonatal resuscitation; and 5 (2%) withdrew from the study at the time of birth; thus, leaving 142 (64%) births that met our inclusion criteria. From those, 109 received SSC and early breastfeeding, representing 77% of the included participants. The rest did not receive SSC and early breastfeeding due to significant respiratory distress in the newborn (*n* = 20) and dysmorphic features that required additional evaluation (*n* = 2), representing 14 and 1% of the included participants, respectively. In the remaining 11 (8%) participants, no reason could be found on the chart regarding why they did not receive SSC and early breastfeeding (Fig. 2). The demographic characteristics of the mothers are described in Table 1. No statistical differences were found between the age, gravidity and gestational age at birth between the mothers who received SSC and early breastfeeding, and those who did not.

**Table 1:** Demographic characteristics of the mothers

Variable	Mothers who received SSC and early breastfeeding (*n* = 109)	Mothers who did not receive SSC and early breastfeeding (*n* = 33)	*p* - value
Age (years)	22.1 ± 5.3	23.1 ± 4.4	0.3
Gravidity			
1	53 (49%)	18 (55%)	0.6
2	26 (24%)	5 (15%)	
3	20 (18%)	8 (24%)	
> 3	10 (9%)	2 (6%)	
Gestational age at birth	39.1 ± 1.4	39.4 ± 1.2	0.2

Comparisons between groups were made through unpaired T-tests for continuous variables and Fisher’s exact tests for categorical data

There was a trend of more mothers receiving SCC as the months of the study passed, albeit it was not statistically significant. The distribution of the births occurred as follows: 45 in the first month, of which 31 (69%) received SSC and early breastfeeding; 55 in the second month, of which 42 (76%) received SSC and early breastfeeding; and 42 in the third month, of which 36 (86%) received SSC and early breastfeeding.

**Fig. 3:**
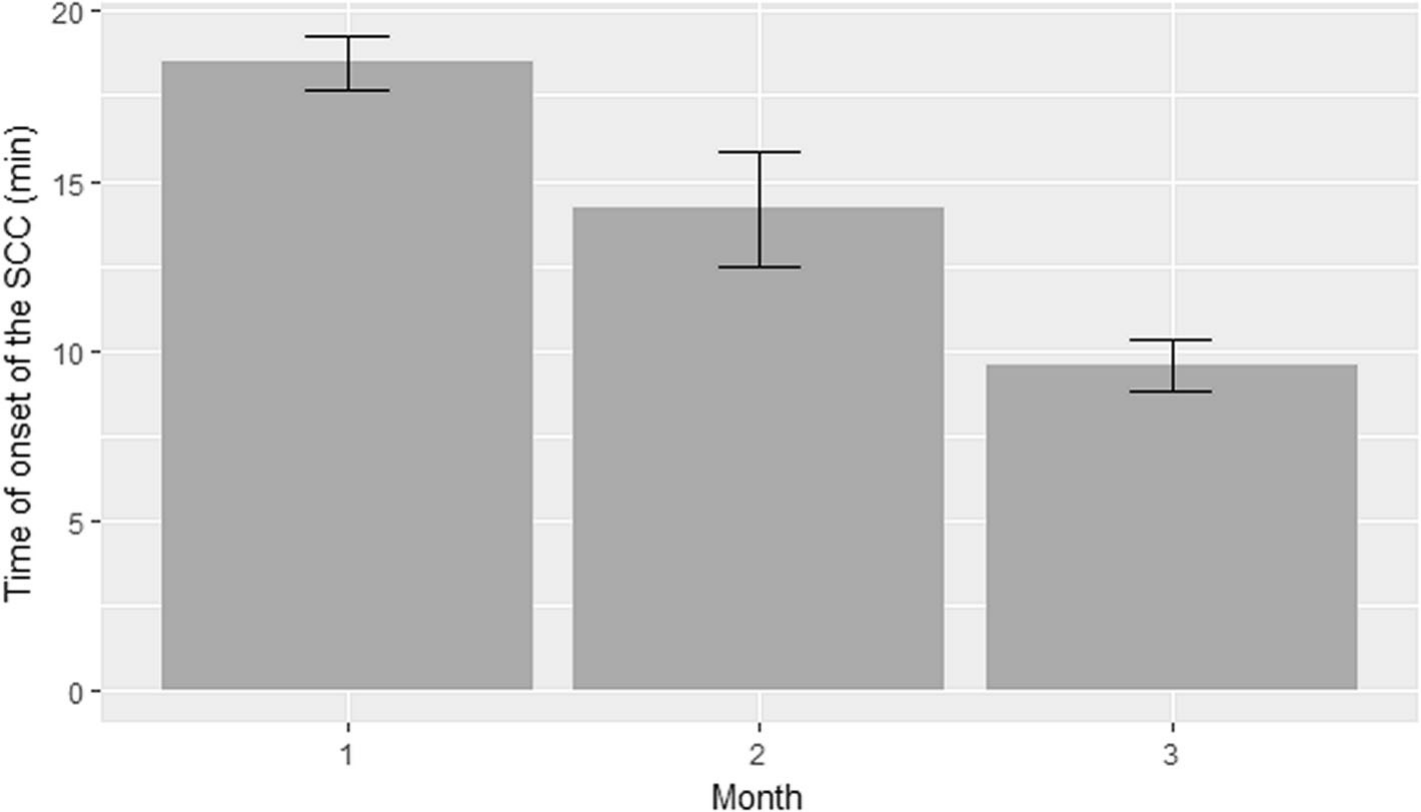
Starting time of skin-to-skin contact. Average starting time of skin-to-skin contact in the first, second and third months of the study was 18.5 (± 2.2), 14.2 (± 5.4) and 9.6 (± 2.2) minutes of life, respectively. A statistically significant difference was found between all groups (*p* < 0 .001)

The average time of SSC initiation in the first, second and third months of the study was 18.5 (± 2.2), 14.2 (± 5.4) and 9.6 (± 2.2) minutes of life, respectively (Fig. 3). There was a statistically significant difference between all groups (*p* < 0.001 in all cases).

**Fig. 4:**
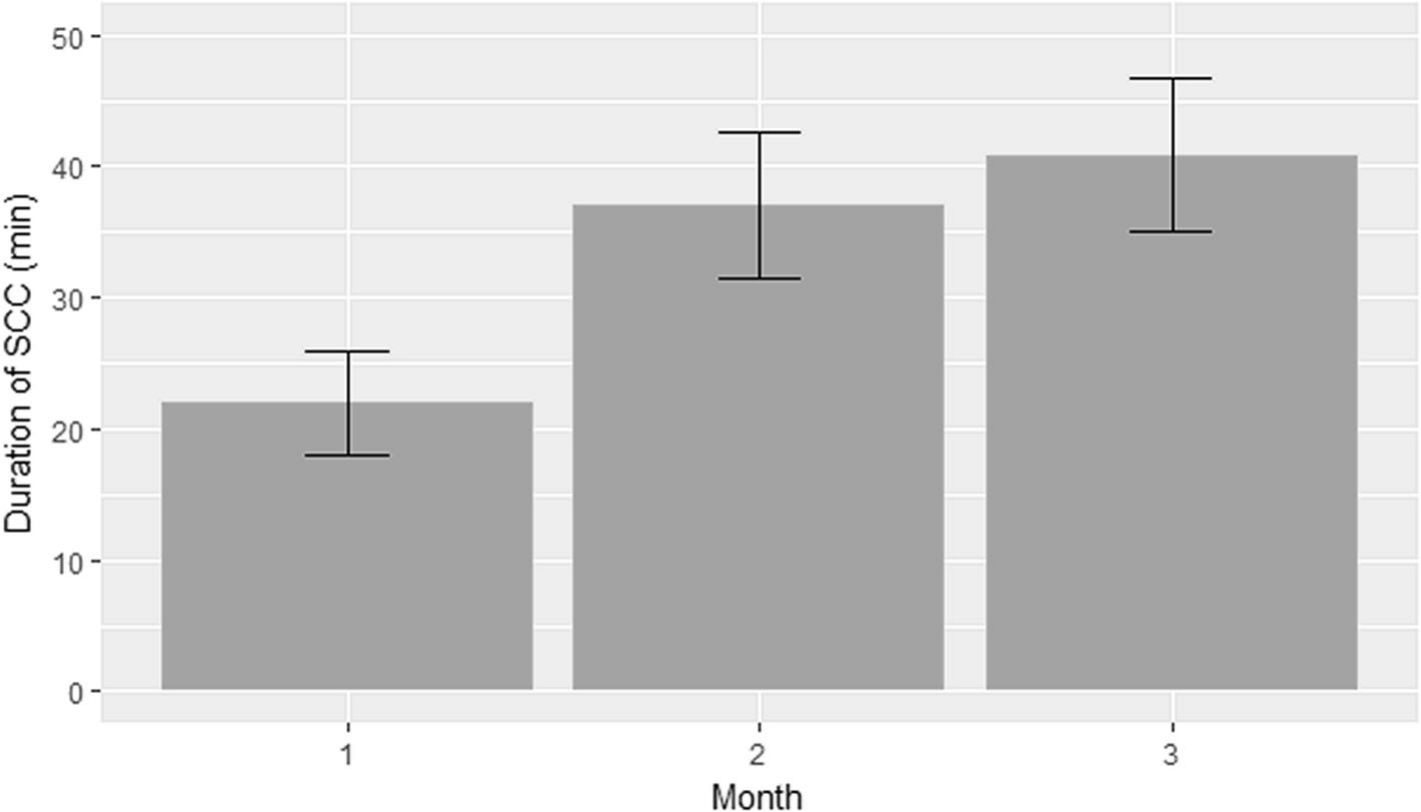
Duration of skin-to-skin contact. Average duration of skin-to-skin contact in the first, second and third months of the study was 22 (± 10.9), 37 (± 17.9) and 40.9 (± 17.4) minutes, respectively. A statistically significant difference was found between the first and second months (*p* < 0.001); and between the first and third months (*p* < 0.001); but not between the second and third months (*p* = 0.540)

The average duration of SSC in the first, second and third months of the study was 22 (± 10.9), 37 (± 17.9) and 40.9 (± 17.4) minutes, respectively (Fig. 4). A statistically significant difference was found between months 1 and 2 (*p* < 0.001), and between months 1 and 3 (*p* < 0.001). There was no significant difference between months 2 and 3 (*p* = 0.540).

**Fig. 5:**
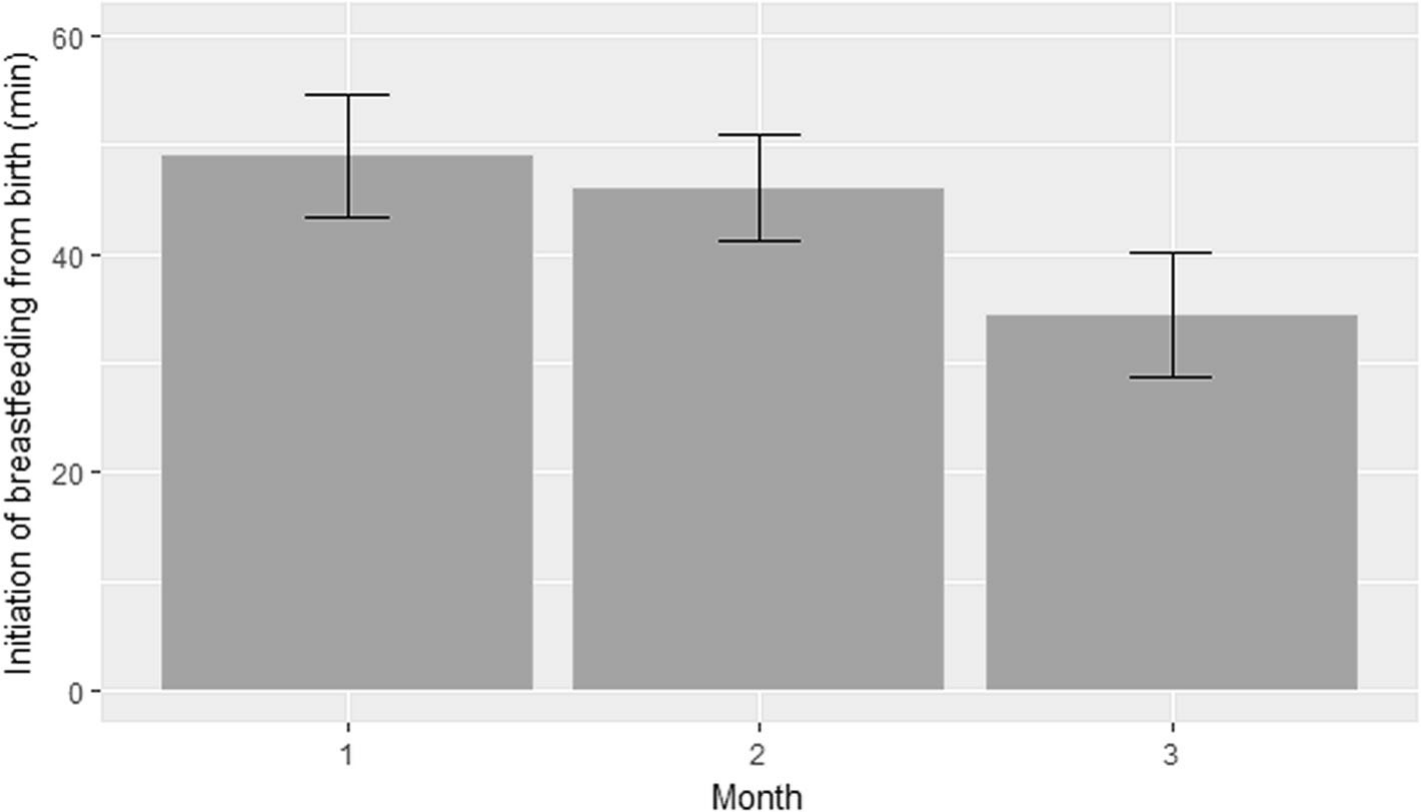
Starting time of breastfeeding. Average starting time of breastfeeding in the first, second and third months of the study was 48.9 (± 15), 45.9 (± 15.5) and 34.4 (± 16.7) minutes of life, respectively. A statistically significant difference was found between the first and third months (*p* < 0.001); and between the second and third months (*p* = 0.005); but not between the first and second months (*p* = 0.705)

The average time of onset of breastfeeding in the first, second and third months of the study was 48.9 (± 15), 45.9 (± 15.5) and 34.4 (± 16.7) minutes of life, respectively (Fig. 5). A statistically significant difference was found between months 1 and 3 (*p* < 0.001) and between months 2 and 3 (*p* = 0.005). There was no statistically significant difference between months 1 and 2 (*p* = 0.705).

ANOVA tests were performed to assess the impact of potential confounding factors, finding that only the month of the study was significantly associated with the time of onset and duration of SSC, and the time of initiation of breastfeeding. The age of the mother, the gravidity and the gestational age at birth were not associated with the time of onset and duration of SSC, nor the time of breastfeeding initiation.

## Discussion

Despite the short period of the study, it was surprising to find that SSC and early breastfeeding were achieved in 77% of the cases (*n* = 109 out of the 142 mothers that consented), especially since it was an intervention that had never been performed in the hospital’s history. One of the reasons why SSC and early breastfeeding had not been implemented before was the lack of knowledge amongst the hospital’s personnel about SSC and its benefits. From the 23% (*n* = 33) who did not receive SSC and early breastfeeding, 22 required further assessment either because they had respiratory distress or were infants with dysmorphic features. This left 11 infants (8% from the total of eligible newborns) that could have received SSC and early breastfeeding but didn’t, representing the group where apparently the intervention had no effect. There was no information on the chart regarding why they did not receive SSC or early breastfeeding.

Throughout the study, the time of initiation of SSC and breastfeeding was reduced progressively and significantly. By the end of the study the goal of immediate SSC (within the first ten minutes of life) was achieved (9.6 ± 2.2 min) [2]. Likewise, the mean time of initiation of breastfeeding by the end of the study was 34.4 (± 16.7) minutes, slightly above the Official Mexican Standard NOM-007 recommendation of starting breastfeeding within the first 30 min of life [9]. Although the minimum duration time in SSC stipulated by the World Health Organization of one hour was not achieved [16], its duration increased significantly across the study, going from 22 (± 10.9) to 40.9 (± 17.4) minutes. These positive trends could be explained by the gradual familiarity the labor and birthing staff obtained over time by performing SSC and early breastfeeding routinely. These findings were similar to previous studies [18, 19], where it was found that health professionals who had the opportunity to practice newly learned skills can modify their professional practice.

It is worth mentioning a phenomenon that spontaneously developed within the nursing staff during the study. At the beginning, after SSC had been started and when the mother had to be transferred from the birthing bed to the recovery bed, the newborn was detached from the mother, placed in the radiant crib, tucked in a warm cloth and once the mother was transferred to the recovery area, the newborn was taken to the mother for breastfeeding initiation. Trained fellow nurses noticed these newborns were having interrupted SSC, and began to correct those nurses who were not following the proposed steps. They supported each other to increase their confidence in transferring the mother and newborn in tandem, thus, preventing SSC from being interrupted. This could explain why the duration of SSC in the first month was so discordant with the time of initiation of breastfeeding. Mistakes were corrected along the way without the intervention of the research team as the conduct was independently modified by the nurses themselves.

In contrast to previous studies [20–23], the number of pregnancies did not affect the breastfeeding starting time. This could be explained by our own cultural setting where SSC and early breastfeeding was something new to our population as well. Even experienced mothers within our setting would be accustomed to breastfeeding their babies for the first time until they were in the maternity ward hours after birth and without SSC. Furthermore, the absence of midwives in our hospital could also be an influencing factor that affected the initiating time of breastfeeding.

Even though similar studies have shown that SSC and early breastfeeding can be implemented through health personnel training [24], to the best of our knowledge, no studies regarding educational interventions promoting SSC and early breastfeeding in rural areas could be identified. More studies need to focus on rural communities since breastfeeding has been described to be a behavior with positive outcomes especially among lower income populations [25].

Finally, it is important to emphasize that promoting breastfeeding and SSC is an easy and cost-effective intervention with positive social and economic impacts [25]. Simple educational interventions, such as ours, can improve the quality and standards of care [26] that help change outdated practices and enhance the knowledge of SSC and breastfeeding in rural settings. Similar interventions can be easily replicated to help meet the World Health Assembly target of more than 50% of infants being breastfed by 2025 [27], making SSC and early breastfeeding key interventions to achieve this goal.

After the results were presented to the study hospital authorities, the department of Quality of Care integrated SSC and early breastfeeding as routine interventions making them the new standard of care.

### Study limitations

This study has several limitations. Firstly, and most important, the short time of the study. A longer study period could have showed if the trends of the measured variables continued to improve, reached a plateau or declined over time. Secondly, we did not evaluate the exact number of newborns who did not achieve continuous and uninterrupted SSC, nor problems related to breastfeeding or difficulties with performing and maintaining SSC. Thirdly, the long term impact of SSC in the mother and the baby was not assessed and the impact of SSC and early breastfeeding on long term breastfeeding after discharge was not analyzed. Fourthly, we did not explore the reasons why those newborns that could receive SCC and early breastfeeding. Lastly, the study was designed to tackle a specific problem found in our hospital’s culture. Although this intervention could be replicated in hospitals with similar outdated practices, its replicability would be limited in different settings.

A follow up study is being planned to evaluate the long-lasting effects of our intervention focusing on: the sustainability of the educational program; the mother’s perceptions and perceived limitations of the intervention; and the impact on breastfeeding during the first six months of life.

## Conclusions

This study demonstrates how a simple and low-cost educational intervention can promote SSC and early breastfeeding in a rural setting by modifying erroneous practices and improving hospital quality of care. Healthcare providers can engage in similar educational interventions that impact their population by bringing the benefits of SSC and early breastfeeding to their communities, no matter how small or remote. Further research regarding educational interventions to promote SSC and early breastfeeding in rural areas is warranted.

## Abbreviations

SSC: skin-to-skin contact
NOM-007: Official Mexican Standard NOM-007-SSA2–2016i
